# 4-Chloro-*N*-(4-chloro­phenyl­sulfon­yl)-*N*-(3-oxo-2,3-dihydro-1,2-benzisothia­zol-2-yl)benzene­sulfonamide

**DOI:** 10.1107/S1600536809003195

**Published:** 2009-01-31

**Authors:** Corrado Rizzoli, Paola Vicini, Matteo Incerti

**Affiliations:** a Dipartimento di Chimica Generale ed Inorganica, Chimica Analitica, Chimica Fisica, Viale G. P. Usberti 17/A, Universitá di Parma, I-43100 Parma, Italy; b Dipartimento Farmaceutico, Viale G. P. Usberti 27/A, Universitá di Parma, I-43100 Parma, Italy

## Abstract

In the title compound, C_19_H_12_Cl_2_N_2_O_5_S_3_, the benzene rings of the chloro­phenyl­sulfonyl groups form a dihedral angle of 35.85 (8)° and are inclined at angles of 23.51 (6) and 59.22 (6)° with respect to the essentially planar benzisothia­zole ring system [maximum deviation = 0.030 (2) Å]. The mol­ecular conformation is stabilized by an intra­molecular C—H⋯O hydrogen bond. In the crystal packing, mol­ecules are linked into chains parallel to the *a* axis by inter­molecular C—H⋯O hydrogen bonds and π–π stacking inter­actions, with centroid–centroid distances of 3.592 (5) Å.

## Related literature

For the synthesis and biological activity of 1,2-benzisothia­zol-3(2*H*)-ones and 2-amino-1,2-benzisothia­zol-3(2*H*)-one derivatives, see: Clerici *et al.* (2007 ▶); Siegemund *et al.* (2002 ▶); Vicini *et al.* (1997 ▶). For the synthesis of the title compound, see: Vicini *et al.* (2009 ▶). For the crystal structures of related benzisothia­zole compounds, see: Cavalca *et al.* (1970 ▶); Ranganathan *et al.* (2002 ▶); Steinfeld & Kersting (2006 ▶); Kim *et al.* (1996 ▶); Xu *et al.* (2006 ▶); Sarma & Mugesh (2007 ▶); Kolberg *et al.* (1999 ▶).
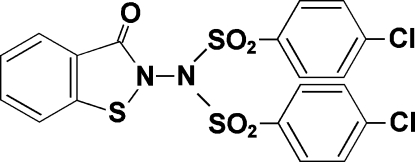


## Experimental

### 

#### Crystal data

C_19_H_12_Cl_2_N_2_O_5_S_3_
*M* *_r_* = 515.39
Triclinic, 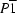

*a* = 9.5358 (12) Å
*b* = 10.7757 (14) Å
*c* = 11.0393 (14) Å
α = 102.719 (2)°
β = 94.385 (3)°
γ = 105.598 (2)°
*V* = 1054.6 (2) Å^3^
*Z* = 2
Mo *K*α radiation
μ = 0.64 mm^−1^
*T* = 295 (2) K
0.22 × 0.14 × 0.12 mm

#### Data collection

Bruker SMART 1000 CCD area-detector diffractometer
Absorption correction: multi-scan (*SADABS*; Bruker, 1997 ▶) *T*_min_ = 0.872, *T*_max_ = 0.927
10953 measured reflections
3930 independent reflections
2267 reflections with *I* > 2σ(*I*)
*R*_int_ = 0.037

#### Refinement

*R*[*F* ^2^ > 2σ(*F* ^2^)] = 0.035
*wR*(*F* ^2^) = 0.060
*S* = 0.94
3930 reflections
280 parameters
H-atom parameters constrained
Δρ_max_ = 0.21 e Å^−3^
Δρ_min_ = −0.22 e Å^−3^

### 

Data collection: *SMART* (Bruker, 1997 ▶); cell refinement: *SAINT* (Bruker, 1997 ▶); data reduction: *SAINT*; program(s) used to solve structure: *SIR97* (Altomare *et al.*, 1999 ▶); program(s) used to refine structure: *SHELXL97* (Sheldrick, 2008 ▶); molecular graphics: *ORTEP-3 for Windows* (Farrugia, 1997 ▶) and *SCHAKAL* (Keller, 1997 ▶); software used to prepare material for publication: *SHELXL97* and *PARST95* (Nardelli, 1995 ▶).

## Supplementary Material

Crystal structure: contains datablocks global, I. DOI: 10.1107/S1600536809003195/lh2763sup1.cif

Structure factors: contains datablocks I. DOI: 10.1107/S1600536809003195/lh2763Isup2.hkl

Additional supplementary materials:  crystallographic information; 3D view; checkCIF report

## Figures and Tables

**Table 1:** Hydrogen-bond geometry (Å, °)

*D*—H⋯*A*	*D*—H	H⋯*A*	*D*⋯*A*	*D*—H⋯*A*
C13—H13⋯O1	0.93	2.42	3.275 (4)	153
C5—H5⋯O2^i^	0.93	2.58	3.353 (4)	140
C6—H6⋯O3^ii^	0.93	2.58	3.289 (3)	133

Symmetry codes: (i) 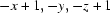; (ii) 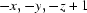.

**Fig. 1.**
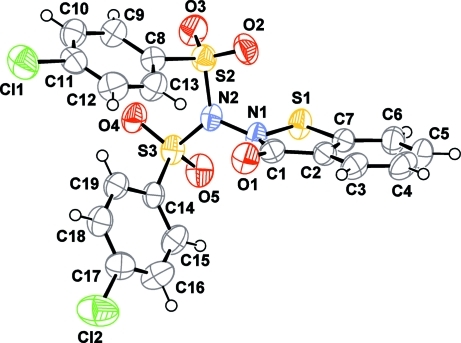
The molecular structure of the title compound, with displacement ellipsoids drawn at the 50% probability level.

**Fig. 2.**
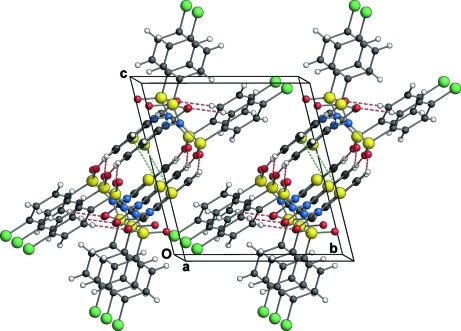
Crystal packing of the title compound viewed approximately along the a axis. Red and green dashed lines indicate C–H···O hydrogen bonds and π-π stacking interactions, respectively.

## supplementary crystallographic information

### Comment

Among 1,2-benzisothiazol-3(2*H*)-ones, a class of compounds with a wide spectrum of biological activities (Clerici *et al.*, 2007; Siegemund *et al.*, 2002), 2-amino-1,2-benzisothiazol-3(2*H*)-one derivatives have a rather recent history and 2-amino-1,2-benzisothiazol-3(2*H*)-one was first synthesized by our group in 1997 (Vicini *et al.*, 1997). Due to their peculiar reactivity, 2-amino-1,2-benzisothiazol-3(2*H*)-one derivatives have recently emerged as effective antiplatelet, spasmolytic and antimicrobial agents (Clerici *et al.*, 2007; Siegemund *et al.*, 2002). The therapeutic significance of the 2-amino-1,2-benzisothiazol-3(2*H*)-one ring system with suitably functionalized substituents has encorauged us to develop novel compounds. The title compound was obtained unintentionally as a by-product during the synthesis of 2-(benzenesulfonyl)amino-1,2-benzisothiazol-3(2*H*)-ones that have been demonstrated to possess anti-HIV-1 activity against wild type virus and against viral strains carrying clinically relevant mutations (Vicini *et al.*, 2008). The unexpected 2-(bisphenylsulfonyl)amino-1,2-benzisothiazol-3(2*H*)-ones, subjected to biological evaluation as well, resulted fairly active and, interestingly, endowed with lower cytotoxicity with respect to their monophenylsulfonyl substituted counterparts. In view of the structure-activity relationship study of the novel 1,2-benzisothiazol-3(2*H*)-one benzenesulfonamides aimed at optimizing their antiretroviral potency, the representative title compound was synthesized and its crystal structure is reported here.

The molecular structure of the title compound is shown in Fig. 1. The bond lengths and angles are unexceptional. The S1—N1 and S1—C7 bond distances within the benzoisothiazole ring system are 1.7347 (19) and 1.744 (2) Å respectively, in good agreement with those reported is related compounds (Cavalca *et al.*, 1970; Ranganathan *et al.*, 2002; Steinfeld & Kersting, 2006; Kim *et al.*, 1996; Xu *et al.*, 2006; Sarma & Mugesh, 2007). The N1—N2 bond distance (1.381 (2) Å) is not significantly different from the corresponding distance in 4,5-dimethyl-2-(3-nitrobenzenesulfonylamino)isothiazol-3(2*H*)-one 1,1-dioxide (1.387 (4) Å; Kolberg *et al.*, 1999). The C8–C13 and C14–C19 benzene rings form a dihedral angle of 35.85 (8)° and are tilted by 23.51 (6) and 59.22 (6)° with respect to the essentially planar benzoisothiazole rings system (maximum deviation 0.030 (2) Å for atom N1). The molecular structure is stabilized by an intramolecular C—H···O hydrogen bond (Table 1). In the crystal packing (Fig. 2), molecules are linked into chains running parallel to the *a* axis by intermolecular C—H···O hydrogen interactions (Table 1) and π-π stacking interactions occurring between the benzene rings of centrosymmetrically related benzisothiazole rings, with a centroid-to-centroid separation of 3.592 (5) Å, a perpendicular interplanar distance of 3.514 (5) Å and a centroid-centroid offset of 0.746 (4) Å (symmetry code linking the adjacent rings: 1 - *x*, -*y*, 1 - *z*).

### Experimental

The title compound was synthesized by reaction of 2-amino-1,2-benzisothiazol-3(2*H*)-one (10 mmol) with 4-chlorobenzenesulfonyl chloride (11 mmol) in pyridine (8 ml) for 2 h at 273K, resulting in a mixture of 4-chloro-*N*-(3-oxo-1,2-benzisothiazol-2(3*H*)-yl)benzenesulfonamide and 4-chloro-*N*-[(4-chlorophenyl)sulfonyl]-*N*-(3-oxo-1,2-benzisothiazol-2(3*H*)-yl)benzenesulfonamide (% yield ratio 33/66). Indeed, once the monophenylsulfonyl product is formed, a subsequent sulfonylation yielding the bisphenylsulfonyl derivative readily occurs, by the action of the electrophilic benzenesulfonyl chloride. The two products were simply separated because of the acidic character of the former. The crude product was poured into water (30 ml) and treated with a 10% aqueous sodium carbonate under stirring for 1 h, affording the title compound as insoluble solid that was collected by filtration. Pale yellow crystals suitable for X-ray analysis were obtained on slow evaporation of an ethanol solution at room temperature.

### Refinement

All H atoms were placed at calculated positions and refined in the riding model approximation, with C—H = 0.93 Å, and with *U*_iso_(H) = 1.2 *U*_eq_(C).

### Crystal data

**Table:**

C_19_H_12_Cl_2_N_2_O_5_S_3_	*Z* = 2
*M**_r_* = 515.39	*F*(000) = 524
Triclinic, *P*1	*D*_x_ = 1.623 Mg m^−^^3^
Hall symbol: -P 1	Mo *K*α radiation, λ = 0.71073 Å
*a* = 9.5358 (12) Å	Cell parameters from 1477 reflections
*b* = 10.7757 (14) Å	θ = 4.8–47.8°
*c* = 11.0393 (14) Å	µ = 0.64 mm^−^^1^
α = 102.719 (2)°	*T* = 295 K
β = 94.385 (3)°	Prism, pale yellow
γ = 105.598 (2)°	0.22 × 0.14 × 0.12 mm
*V* = 1054.6 (2) Å^3^	

### Data collection

**Table:**

Bruker SMART 1000 CCD area-detector diffractometer	3930 independent reflections
Radiation source: fine-focus sealed tube	2267 reflections with *I* > 2σ(*I*)
graphite	*R*_int_ = 0.037
ω scans	θ_max_ = 25.5°, θ_min_ = 1.9°
Absorption correction: multi-scan (*SADABS*; Bruker, 1997)	*h* = −11→11
*T*_min_ = 0.872, *T*_max_ = 0.927	*k* = −13→13
10953 measured reflections	*l* = −13→13

### Refinement

**Table:**

Refinement on *F*^2^	Primary atom site location: structure-invariant direct methods
Least-squares matrix: full	Secondary atom site location: difference Fourier map
*R*[*F*^2^ > 2σ(*F*^2^)] = 0.035	Hydrogen site location: inferred from neighbouring sites
*wR*(*F*^2^) = 0.060	H-atom parameters constrained
*S* = 0.94	*w* = 1/[σ^2^(*F*_o_^2^) + (0.0145*P*)^2^] where *P* = (*F*_o_^2^ + 2*F*_c_^2^)/3
3930 reflections	(Δ/σ)_max_ = 0.001
280 parameters	Δρ_max_ = 0.21 e Å^−^^3^
0 restraints	Δρ_min_ = −0.21 e Å^−^^3^

### Special details

**Table:**

Refinement. Refinement of *F*^2^ against ALL reflections. The weighted *R*-factor *wR* and goodness of fit *S* are based on *F*^2^, conventional *R*-factors *R* are based on *F*, with *F* set to zero for negative *F*^2^. The threshold expression of *F*^2^ > σ(*F*^2^) is used only for calculating *R*-factors(gt) *etc*. and is not relevant to the choice of reflections for refinement. *R*-factors based on *F*^2^ are statistically about twice as large as those based on *F*, and *R*- factors based on ALL data will be even larger.

### Fractional atomic coordinates and isotropic or equivalent isotropic displacement parameters (Å^2^)

**Table:**

	*x*	*y*	*z*	*U*_iso_*/*U*_eq_	
Cl1	0.30528 (10)	0.89637 (7)	0.94340 (8)	0.0862 (3)	
Cl2	0.37123 (10)	0.47508 (9)	1.37354 (8)	0.1051 (3)	
S1	0.13674 (7)	−0.02399 (6)	0.62308 (7)	0.0550 (2)	
S2	0.14511 (8)	0.31023 (7)	0.62932 (7)	0.0539 (2)	
S3	0.01047 (7)	0.21135 (7)	0.84324 (7)	0.0515 (2)	
O1	0.43050 (18)	0.27563 (17)	0.83915 (17)	0.0630 (5)	
O2	0.2660 (2)	0.28393 (17)	0.57181 (16)	0.0677 (5)	
O3	0.00450 (19)	0.27802 (16)	0.55643 (16)	0.0687 (6)	
O4	−0.07297 (17)	0.29573 (16)	0.81554 (16)	0.0605 (5)	
O5	−0.05678 (18)	0.07361 (16)	0.83264 (17)	0.0639 (5)	
N1	0.2112 (2)	0.12783 (18)	0.73222 (19)	0.0501 (6)	
N2	0.1337 (2)	0.22006 (18)	0.74166 (18)	0.0476 (5)	
C1	0.3647 (3)	0.1683 (3)	0.7692 (2)	0.0478 (7)	
C2	0.4188 (3)	0.0593 (2)	0.7077 (2)	0.0451 (6)	
C3	0.5641 (3)	0.0591 (3)	0.7197 (2)	0.0554 (7)	
H3	0.6367	0.1309	0.7718	0.066*	
C4	0.5991 (3)	−0.0492 (3)	0.6532 (3)	0.0654 (8)	
H4	0.6964	−0.0507	0.6600	0.078*	
C5	0.4911 (3)	−0.1560 (3)	0.5762 (3)	0.0638 (8)	
H5	0.5174	−0.2288	0.5330	0.077*	
C6	0.3462 (3)	−0.1582 (3)	0.5614 (2)	0.0575 (7)	
H6	0.2744	−0.2306	0.5090	0.069*	
C7	0.3110 (3)	−0.0472 (2)	0.6283 (2)	0.0462 (6)	
C8	0.1916 (3)	0.4758 (2)	0.7178 (2)	0.0477 (7)	
C9	0.0978 (3)	0.5504 (3)	0.7018 (2)	0.0560 (7)	
H9	0.0109	0.5132	0.6456	0.067*	
C10	0.1350 (3)	0.6819 (3)	0.7707 (3)	0.0611 (8)	
H10	0.0739	0.7342	0.7603	0.073*	
C11	0.2626 (3)	0.7341 (2)	0.8544 (2)	0.0561 (7)	
C12	0.3557 (3)	0.6602 (3)	0.8706 (3)	0.0657 (8)	
H12	0.4419	0.6976	0.9275	0.079*	
C13	0.3203 (3)	0.5293 (3)	0.8017 (3)	0.0614 (8)	
H13	0.3826	0.4779	0.8117	0.074*	
C14	0.1174 (2)	0.2839 (2)	0.9908 (2)	0.0450 (6)	
C15	0.1572 (3)	0.2053 (3)	1.0622 (3)	0.0630 (8)	
H15	0.1308	0.1134	1.0312	0.076*	
C16	0.2365 (3)	0.2648 (3)	1.1799 (3)	0.0772 (9)	
H16	0.2630	0.2130	1.2295	0.093*	
C17	0.2764 (3)	0.4011 (3)	1.2242 (3)	0.0632 (8)	
C18	0.2397 (3)	0.4797 (3)	1.1525 (3)	0.0593 (8)	
H18	0.2694	0.5718	1.1826	0.071*	
C19	0.1583 (3)	0.4206 (2)	1.0355 (3)	0.0531 (7)	
H19	0.1309	0.4726	0.9866	0.064*	

### Atomic displacement parameters (Å^2^)

**Table:**

	*U*^11^	*U*^22^	*U*^33^	*U*^12^	*U*^13^	*U*^23^
Cl1	0.1137 (7)	0.0495 (5)	0.0768 (6)	0.0059 (4)	0.0144 (5)	−0.0001 (4)
Cl2	0.1116 (7)	0.1090 (7)	0.0712 (6)	0.0070 (6)	−0.0208 (5)	0.0175 (5)
S1	0.0427 (4)	0.0480 (4)	0.0663 (5)	0.0159 (3)	−0.0044 (4)	−0.0012 (4)
S2	0.0533 (5)	0.0505 (5)	0.0519 (5)	0.0148 (4)	0.0004 (4)	0.0035 (4)
S3	0.0382 (4)	0.0465 (4)	0.0652 (5)	0.0107 (3)	0.0072 (4)	0.0067 (4)
O1	0.0498 (11)	0.0554 (12)	0.0689 (13)	0.0092 (10)	−0.0101 (10)	−0.0007 (10)
O2	0.0727 (13)	0.0704 (13)	0.0658 (13)	0.0290 (11)	0.0304 (11)	0.0121 (10)
O3	0.0659 (12)	0.0604 (12)	0.0646 (13)	0.0196 (10)	−0.0240 (10)	−0.0055 (10)
O4	0.0441 (10)	0.0671 (12)	0.0743 (13)	0.0273 (10)	0.0043 (9)	0.0136 (10)
O5	0.0517 (11)	0.0423 (11)	0.0842 (14)	−0.0009 (9)	0.0102 (10)	0.0064 (10)
N1	0.0398 (13)	0.0440 (13)	0.0617 (15)	0.0170 (11)	0.0009 (11)	−0.0002 (11)
N2	0.0433 (12)	0.0427 (13)	0.0591 (14)	0.0168 (10)	0.0119 (11)	0.0108 (11)
C1	0.0416 (16)	0.0511 (18)	0.0499 (18)	0.0127 (14)	0.0007 (14)	0.0139 (14)
C2	0.0411 (16)	0.0508 (17)	0.0444 (17)	0.0155 (14)	0.0053 (13)	0.0119 (13)
C3	0.0402 (16)	0.0617 (19)	0.068 (2)	0.0166 (14)	0.0050 (15)	0.0228 (16)
C4	0.0469 (18)	0.081 (2)	0.086 (2)	0.0333 (18)	0.0151 (17)	0.0384 (19)
C5	0.065 (2)	0.067 (2)	0.072 (2)	0.0377 (18)	0.0179 (18)	0.0175 (17)
C6	0.0581 (19)	0.0574 (19)	0.0596 (19)	0.0262 (15)	0.0077 (15)	0.0086 (15)
C7	0.0459 (16)	0.0517 (17)	0.0457 (17)	0.0204 (14)	0.0083 (14)	0.0136 (14)
C8	0.0442 (16)	0.0445 (16)	0.0512 (17)	0.0087 (13)	0.0069 (14)	0.0111 (13)
C9	0.0483 (17)	0.0503 (18)	0.065 (2)	0.0112 (14)	0.0023 (15)	0.0121 (15)
C10	0.0604 (19)	0.0529 (19)	0.071 (2)	0.0164 (16)	0.0114 (17)	0.0163 (16)
C11	0.0647 (19)	0.0431 (17)	0.0545 (19)	0.0032 (15)	0.0185 (16)	0.0121 (14)
C12	0.0598 (19)	0.056 (2)	0.064 (2)	0.0015 (16)	−0.0087 (16)	0.0054 (16)
C13	0.0516 (17)	0.0563 (19)	0.071 (2)	0.0160 (15)	−0.0043 (16)	0.0104 (16)
C14	0.0384 (15)	0.0426 (16)	0.0527 (17)	0.0126 (13)	0.0082 (13)	0.0080 (14)
C15	0.065 (2)	0.0434 (17)	0.081 (2)	0.0162 (15)	0.0061 (18)	0.0180 (17)
C16	0.079 (2)	0.069 (2)	0.086 (3)	0.0211 (19)	−0.006 (2)	0.031 (2)
C17	0.0556 (18)	0.068 (2)	0.062 (2)	0.0124 (16)	0.0010 (15)	0.0160 (17)
C18	0.0550 (18)	0.0442 (17)	0.070 (2)	0.0097 (14)	0.0032 (16)	0.0049 (16)
C19	0.0511 (17)	0.0461 (18)	0.0614 (19)	0.0136 (14)	0.0074 (15)	0.0134 (15)

### Geometric parameters (Å, °)

**Table:**

Cl1—C11	1.729 (3)	C5—H5	0.9300
Cl2—C17	1.726 (3)	C6—C7	1.399 (3)
S1—N1	1.7347 (19)	C6—H6	0.9300
S1—C7	1.744 (2)	C8—C9	1.379 (3)
S2—O2	1.4214 (17)	C8—C13	1.381 (3)
S2—O3	1.4239 (16)	C9—C10	1.388 (3)
S2—N2	1.729 (2)	C9—H9	0.9300
S2—C8	1.754 (2)	C10—C11	1.371 (3)
S3—O4	1.4240 (16)	C10—H10	0.9300
S3—O5	1.4242 (16)	C11—C12	1.368 (3)
S3—N2	1.684 (2)	C12—C13	1.383 (3)
S3—C14	1.750 (3)	C12—H12	0.9300
O1—C1	1.213 (3)	C13—H13	0.9300
N1—N2	1.381 (2)	C14—C15	1.379 (3)
N1—C1	1.409 (3)	C14—C19	1.381 (3)
C1—C2	1.461 (3)	C15—C16	1.376 (4)
C2—C3	1.383 (3)	C15—H15	0.9300
C2—C7	1.388 (3)	C16—C17	1.377 (4)
C3—C4	1.371 (3)	C16—H16	0.9300
C3—H3	0.9300	C17—C18	1.370 (3)
C4—C5	1.380 (3)	C18—C19	1.377 (3)
C4—H4	0.9300	C18—H18	0.9300
C5—C6	1.371 (3)	C19—H19	0.9300
			
N1—S1—C7	89.10 (11)	C2—C7—S1	113.04 (18)
O2—S2—O3	120.38 (11)	C6—C7—S1	126.2 (2)
O2—S2—N2	102.15 (10)	C9—C8—C13	121.2 (2)
O3—S2—N2	109.77 (11)	C9—C8—S2	118.9 (2)
O2—S2—C8	110.89 (11)	C13—C8—S2	119.9 (2)
O3—S2—C8	108.61 (12)	C8—C9—C10	119.0 (2)
N2—S2—C8	103.62 (11)	C8—C9—H9	120.5
O4—S3—O5	121.61 (11)	C10—C9—H9	120.5
O4—S3—N2	104.10 (10)	C11—C10—C9	119.4 (3)
O5—S3—N2	106.59 (10)	C11—C10—H10	120.3
O4—S3—C14	109.51 (11)	C9—C10—H10	120.3
O5—S3—C14	109.23 (12)	C12—C11—C10	121.6 (3)
N2—S3—C14	104.29 (10)	C12—C11—Cl1	119.2 (2)
N2—N1—C1	120.88 (19)	C10—C11—Cl1	119.1 (2)
N2—N1—S1	117.96 (15)	C11—C12—C13	119.4 (3)
C1—N1—S1	116.69 (17)	C11—C12—H12	120.3
N1—N2—S3	115.94 (15)	C13—C12—H12	120.3
N1—N2—S2	117.41 (15)	C8—C13—C12	119.3 (3)
S3—N2—S2	125.38 (12)	C8—C13—H13	120.4
O1—C1—N1	122.8 (2)	C12—C13—H13	120.4
O1—C1—C2	130.4 (2)	C15—C14—C19	120.8 (2)
N1—C1—C2	106.9 (2)	C15—C14—S3	120.4 (2)
C3—C2—C7	120.6 (2)	C19—C14—S3	118.8 (2)
C3—C2—C1	125.3 (2)	C16—C15—C14	119.1 (3)
C7—C2—C1	114.1 (2)	C16—C15—H15	120.5
C4—C3—C2	118.7 (3)	C14—C15—H15	120.5
C4—C3—H3	120.7	C15—C16—C17	119.9 (3)
C2—C3—H3	120.7	C15—C16—H16	120.0
C3—C4—C5	120.6 (2)	C17—C16—H16	120.0
C3—C4—H4	119.7	C18—C17—C16	121.2 (3)
C5—C4—H4	119.7	C18—C17—Cl2	119.0 (2)
C6—C5—C4	122.1 (3)	C16—C17—Cl2	119.9 (2)
C6—C5—H5	119.0	C17—C18—C19	119.2 (3)
C4—C5—H5	119.0	C17—C18—H18	120.4
C5—C6—C7	117.2 (3)	C19—C18—H18	120.4
C5—C6—H6	121.4	C18—C19—C14	119.9 (3)
C7—C6—H6	121.4	C18—C19—H19	120.1
C2—C7—C6	120.8 (2)	C14—C19—H19	120.1

### Hydrogen-bond geometry (Å, °)

**Table:**

*D*—H···*A*	*D*—H	H···*A*	*D*···*A*	*D*—H···*A*
C13—H13···O1	0.93	2.42	3.275 (4)	153
C5—H5···O2^i^	0.93	2.58	3.353 (4)	140
C6—H6···O3^ii^	0.93	2.58	3.289 (3)	133

Symmetry codes: (i) −*x*+1, −*y*, −*z*+1; (ii) −*x*, −*y*, −*z*+1.
